# Supplementary material for: Photoreceptor genes in a trechine beetle, Trechiama kuznetsovi, living in the upper hypogean zone
Source: Zoological Lett. 2023 May 12;9:9. doi: 10.1186/s40851-023-00208-7 (PMC10176714; doi:10.1186/s40851-023-00208-7)
Supplement: Supplementary file 1 — Additional file 1: Table S1. The result of genome and transcript sequencing in T. kuznetsovi. [file 40851_2023_208_MOESM1_ESM.pdf]

Table S1

	Genomic sequences				Transcript sequences			
	Forward reads		Reverse reads		Forward reads		Reverse reads	
Trimming	Before	After	Before	After	Before	After	Before	After
Total sequences (n)	330,675, 288	327,183, 843	330,675, 288	327,183, 843	31,361, 556	29,595, 932	31,361, 556	29,595, 932
Sequence length (bp)	151	15-150	151	15-150	101	15-100	101	15-100
GC (%)	26	27	27	27	44	44	42	42
