# Supplementary material for: Photoreceptor genes in a trechine beetle, Trechiama kuznetsovi, living in the upper hypogean zone
Source: Zoological Lett. 2023 May 12;9:9. doi: 10.1186/s40851-023-00208-7 (PMC10176714; doi:10.1186/s40851-023-00208-7)
Supplement: Supplementary file 2 — Additional file 2: Table S2. BLAST search for opsin genes to the assembled genome and transcripts in T. kuznetsovi. [file 40851_2023_208_MOESM2_ESM.pdf]

Table S2

Query	Database	Hit	E value	Aligned site
Pcha Lw opsin APY20654.1 (382 aa)	Genome	scaffold53886_cov147	1e-56	75 - 206
		scaffold50064_cov139	5e-47	217 - 382
		scaffold36250_cov150	4e-32	1 - 97
	Transcript	TRINITY_DN15450_c0_g1_i2	0.0	1 - 282
		TRINITY_DN4241_c0_g1_i1	1e-53	278 - 382
Pcha Uv opsin APY20653.1 (385 aa)	Genome	scaffold54798_cov158	4e-103	1 - 341
	Transcript	No hit		
