# Supplementary material for: Photoreceptor genes in a trechine beetle, Trechiama kuznetsovi, living in the upper hypogean zone
Source: Zoological Lett. 2023 May 12;9:9. doi: 10.1186/s40851-023-00208-7 (PMC10176714; doi:10.1186/s40851-023-00208-7)
Supplement: Supplementary file 3 — Additional file 3: Table S3. Primers used in PCR and RT-PCR to amplify opsin genes in T. kuznetsovi. T, annealing temperature. [file 40851_2023_208_MOESM3_ESM.pdf]

Table S3

Target	Template	Forward primers	Reverse primers
lw opsin	gDNA	5’ AAGGTCCGCATTTTCACTTG 3’	5’ TCCTCTAGAAGCGCCTTTTAAT 3’
		5’ TTGCAAATTTTGGCACTCTG 3’	5’ CCAAAATGTAACTTCGGCTCA 3’
		5’ ACGATTCGAAGCAATGATGG 3’	5’ CTAGCCCGTGTCCGATAAAA 3’
			5’ GAAACGATGAAATTAGAG 3’
lw opsin	cDNA	5’ ATGTGCTGTATGTCTCCCGC 3’	5’ CAGTCGTTCCAGAAACTGCG 3’
		5’ TTTGGCACTCTGGGCTTTCT 3’	5’ GTGTCTTCTGGTGGAGGAGG 3’
uv opsin	cDNA	5’ AGGGTTGTTCGGAAAATAGTTG 3’	3sites Adapter Primer (3’ RACE CORE Set)
		5’ CGGGGTGGTTATTTCAAGTC 3’	5’ AATGCTTGATTTCCAAACGC 3’
		5’ ACCTAGCCTTTTGCGACCTT 3’	5’ AGAGAGGTAAAACCGCCCAT 3’
		5’ TTTTCGTCGCGGTAATTTTC 3’	5’ ATCGTGTCCAATCCATAGCC 3’
