# Supplementary material for: Photoreceptor genes in a trechine beetle, Trechiama kuznetsovi, living in the upper hypogean zone
Source: Zoological Lett. 2023 May 12;9:9. doi: 10.1186/s40851-023-00208-7 (PMC10176714; doi:10.1186/s40851-023-00208-7)
Supplement: Supplementary file 4 — Additional file 4: Table S4. BLAST search for opsin genes to non-redundant protein sequences in NCBI database. [file 40851_2023_208_MOESM4_ESM.pdf]

Table S4

Query	Hit	E value	Identity (%)
Tkuz Lw opsin (379 aa)	*Pogonus chalceus* LW APY20654.1	0.0	89.03
	Thermonectus marmoratus LW ACH56536.1	0.0	79.68
	Gyrinus marinus LW1 APY20606.1	0.0	79.68
	Tribolium castaneum LW NP_001155991.1	0.0	79.37
Tkuz Uv opsin (373 aa)	*Pogonus chalceus* UV APY20653.1	0.0	83.71
	Thermonectus marmoratus UV1 ACH56537.1	0.0	78.75
	Thermonectus marmoratus UV2 ACH56538.1	0.0	71.27
	Gyrinus marinus UV1 APY20608.1	0.0	75.60
	Tribolium castaneum UV isoform x1 XP_970344.2	0.0	70.67
